# MicroRNA Expression Profiling in HCV-Infected Human Hepatoma Cells Identifies Potential Anti-Viral Targets Induced by Interferon-α

**DOI:** 10.1371/journal.pone.0055733

**Published:** 2013-02-13

**Authors:** Xiaozhen Zhang, Marybeth Daucher, David Armistead, Rodney Russell, Shyam Kottilil

**Affiliations:** 1 Immunopathogenesis Section, Laboratory of Immunoregulation, National Institute of Allergy and Infectious Diseases, National Institutes of Health, Bethesda, Maryland, United States of America; 2 Life Technologies Incorporated, Gaithersburg, Maryland, United States of America; 3 Faculty of Medicine, Health Sciences Center, Memorial University of Newfoundland, St. John’s, Canada; University of California, Merced, United States of America

## Abstract

**Objective:**

Increasing evidence suggests that miRNAs have a profound impact on host defense to Hepatitis C virus (HCV) infection and clinical outcome of standard HCV therapy. In this study, we investigated modulation of miRNA expression in Huh7.5 hepatoma cells by HCV infection and *in vitro* interferon-αtreatment.

**Methods:**

MiRNA expression profiling was determined using Human miRNA TaqMan® Arrays followed by rigorous pairwise statistical analysis. MiRNA inhibitors assessed the functional effects of miRNAs on HCV replication. Computational analysis predicted anti-correlated mRNA targets and their involvement in host cellular pathways. Quantitative RTPCR confirmed the expression of predicted miRNA-mRNA correlated pairs in HCV-infected Huh7.5 cells with and without interferon-α.

**Results:**

Seven miRNAs (miR-30b, miR-30c, miR-130a, miR-192, miR-301, miR-324-5p, and miR-565) were down-regulated in HCV-infected Huh7.5 cells (p<0.05) and subsequently up-regulated following interferon-α treatment (p<0.01). The miR-30(a-d) cluster and miR-130a/301 and their putative mRNA targets were predicted to be associated with cellular pathways that involve Hepatitis C virus entry, propagation and host response to viral infection.

**Conclusions:**

HCV differentially modulates miRNAs to facilitate entry and early establishment of infection *in vitro*. Interferon-α appears to neutralize the effect of HCV replication on miRNA regulation thus providing a potential mechanism of action in eradicating HCV from hepatocytes.

## Introduction

Chronic Hepatitis C (CHC) is a leading cause of liver disease world-wide [1]. Approximately 170 million people are chronically infected with HCV, and some develop progressive liver disease which may lead to cirrhosis, hepatocellular carcinoma and death [2]. The response to current standard therapy for CHC genotype 1 consists of pegylated interferon-α (IFN-α) and ribivarin and results in only 50% of all treated patients achieving sustained virological response (SVR) [3]–[5]. Furthermore, IFN-ribavirin treatment is associated with substantial cost and significant side effects, which has prompted the search for complementary or alternative clinical intervention. A thorough understanding of the underlying pathogenic mechanisms associated with therapeutic response would be critical in optimizing therapy for these individuals. Recent studies have implicated interferon stimulated gene expression [6]–[7] and microRNA (miRNA) expression as factors associated with unfavorable therapeutic outcomes [8]–[10].

MiRNAs are a family of small non-coding RNAs that have emerged as important regulators of gene expression at the post-transcriptional level in a sequence specific manner [11]–[13]. Many miRNAs are evolutionarily conserved and believed to play a role in controlling a variety of biological functions, including developmental patterning, cell differentiation, cell proliferation, genome rearrangements and transcriptional regulation [14]. Increasing evidence has suggested the involvement of miRNAs in viral infection [15]. MiRNAs regulate gene expression largely by degrading target mRNAs; those transcripts whose expression levels are concomitantly decreased are potential targets of concurrently up-regulated miRNAs. Conversely, up-regulated genes may be influenced by miRNAs whose levels are lower. MiRNA target prediction algorithms [16]–[18] and gene ontology databases can be used to refine a list of candidate mRNA targets in large expression datasets and predict the consequence of miRNA expression in cellular pathways. In this study, we performed comprehensive analysis of miRNA profiling using an HCV continuous culture infection system to identify novel miRNAs involved in HCV infection and response to IFN-α. Seven miRNAs were identified as being differentially regulated in HCV infection and following IFN-α treatment *in vitro*. We found that miRNA 30(a–d) and miR-130a/301 share mRNA targets in biological pathways that interact directly with viral entry and propagation. Our results suggest that miRNAs are likely to be involved in regulating a network of molecular and cellular gene functions during HCV infection that are reversed following interferon treatment. This information may illuminate the potential use of miRNAs in the pathogenesis of chronic HCV infection and potential biological mechanisms of interferon α treatment.

## Methods

### HCV Infection and Interferon Treatment of Huh7.5 Cells

Huh7.5 parental hepatoma cells containing a full length HCV genotype 2a replicon were obtained from Apath LLC (Saint Louis, Missouri). Huh7.5 cells were cultured in Dulbecco’s modified eagle medium (DMEM; Invitrogen Inc., Gaithersburg, MD) supplemented with 10% FBS and 100U ml-1 penicillin plus 100 ug ml-1 streptomycin at 37°C in a 5% CO2 containing incubator. For *in vitro* HCV infection, approximately 1×10^6^ Huh7.5 cells were plated in 10 cm plates and incubated overnight at 37°C and 5% CO_2_. Cells were infected with 1.6×10^7^ copies of HCV genotype 2a viral supernatant for 4 hrs at 37°C. Following infection, the medium was replaced with fresh supplemented DMEM thus initiating the Huh7.5 continuous culture system. Three days post-infection, infected cells were seeded in 6 well plates at 2.5×10^5^ cells per well, rested overnight at 37C, 5% CO_2_, and harvested for total RNA the following day. The remaining HCV- infected cells were split one to two times a week for a total of 8 weeks. Cellular RNA was extracted from 4 consecutive cell passages. For HCV plus interferon experiments, HCV^+^Huh7.5 cells from each passage were treated with 50 International Units (IU) ml-1 of IFN-α 2b for 24 hrs. Cells were then trypsinized, washed and prepared for miRNA array analysis.

### MicroRNA Expression Profiling using the Human Taqman® Array

Total RNA purified from each passage of uninfected, HCV-infected and IFN-α -treated HCV-infected Huh7.5 cells (mirVAna miRNA isolation kit, Applied Biosystems, Inc., Foster City, CA) was applied to the TaqMan® Array Human MicroRNA Panel v1.0 using the manufacturer’s suggested protocols (Applied Biosystems, Inc.). A default threshold of 0.2 ΔRn units and an automatic baseline algorithm were applied to the miRNA array data. Fold changes in miRNA expression were calculated using the comparative Ct method [19] and normalized to the RNU44 endogenous control. Pairwise t-tests were performed on all combinations of samples to identify differentially expressed miRNAs at 0.05 and 0.01 confidence. P values from the t-test were used to calculate False Discovery Rates (FDR) using the Benjamini-Hochberg method. Log_2_ fold changes (ddCT) for detected assays were calculated and samples clustered based on differential miRNA expression and Euclidean distances.

### Quantitative RT-PCR Validation of Expression Arrays

Two-step quantitative RT-PCR (q-RTPCR) was performed to validate miRNA expression data. The RT-PCR method was based on a simple two-step process using stem-loop technology for reverse transcription (RT) of the mature microRNA followed by quantitative real-time PCR (Applied Biosystems, Inc). Total RNA was reverse transcribed using specific miRNA primers and the TaqMan® MicroRNA Reverse Transcription Kit followed by cDNA amplification using the TaqMan® MicroRNA Assay (Applied Biosystems, Inc.) Results were analyzed using the comparative Ct method for the relative quantitation of miRNA expression. Small Non-coding RNA RNU44 was used as an endogenous control for all amplifications.

### Transfection of miRNA Inhibitors

Selected miRNA Anti-miRs (inhibitor) were synthesized by Ambion Inc. (Austin, TX). MiRNA inhibitors are single-stranded, modified RNAs which specifically block miRNA function following transfection. Huh7.5 cells were transfected with miRNA Anti-miRs using siPORT NeoFX transfection agent (Ambion, Inc.) then infected with HCV. Mock-transfected HCV infected Huh7.5 cells were used as a negative control. Seventy-two hours post-infection, total RNA extracted from cell lysates and HCV RNA from cell supernatant (QIAmp Viral RNA Extraction Kit, Qiagen, Inc., Germantown, MD). HCV replication was assessed by q-RT PCR and the Mann-Whitney test for multiple comparisons was applied to each experimental condition versus the mock-transfected HCV^+^ Huh7.5 control.

### Bioinformatic Prediction of mRNA Targets and Gene Ontology

MiRNA gene targets were predicted using the JTarget tool from GOmir [20] (http://www.bioacademy.gr/bioinformatics/projects/GOmir/). A list of common gene targets from at least 5 prediction databases (Tarbase, TargetScan, miRanda, RNAhybrid and PicTar (reviewed by [21]) was obtained for each differentially expressed miRNA and applied to the DAVID gene-enrichment and functional annotation analysis database ( [22] http://david.abcc.ncifcrf.gov) to predict gene ontology and biological pathways. Gene lists and background were selected based on expression in humans only. An Expression Analysis Systematic Explorer (EASE) of 0.05, Fisher Exact Test and Benjamini-Hochberg False Discovery Rate (FDR) assessed statistical significance of bio-pathway prediction. TargetScan Human 6.2 (http://www.targetscan.org/) was used to validate predicted miRNA-mRNA targets obtained from DAVID. TargetScan predicts biological targets of miRNAs by searching for conserved 7 mer and 8 mer sites that match the seed region of each miRNA [18]. In mammals, predictions are ranked on predicted efficiency of targeting and by their probability of conserved targeting (P_CT_) [23]. Version 6.2 retrieves regulatory targets of mammalian miRNAs and extends context score contributions to include seed-pairing stability and target-site abundance as well as 3′ UTRs from RefSeq and additional miRNA families [24].

## Results

### Differential miRNA Expression Profiles Associated with HCV

**Figure 1:**
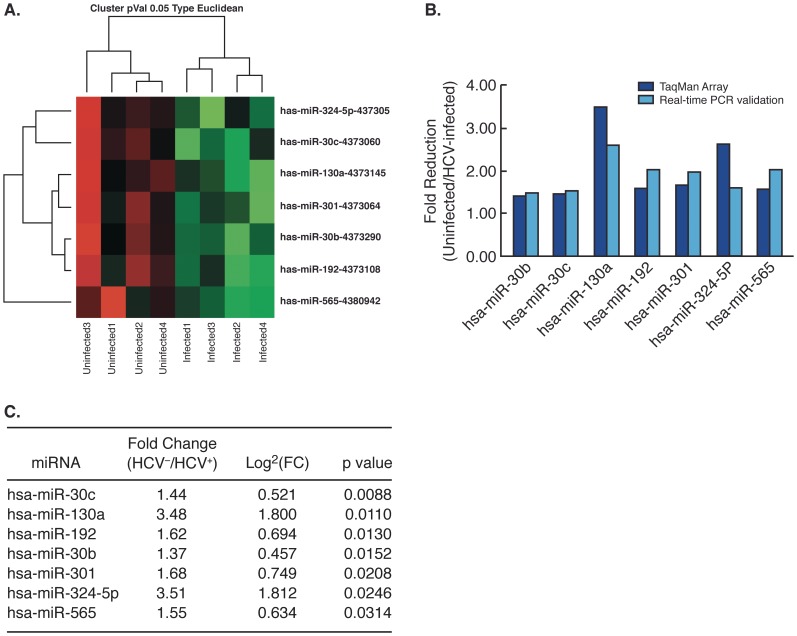
MiRNA expression and validation in HCV-infected Huh7.5 cells. (A) Heat map of differentially expressed miRNAs in uninfected and HCV-infected Huh7.5 cells using a continuous HCV culture system. Red indicates up-regulation of expression and green indicates down-regulation of expression. Seven miRNAs were down-regulated in HCV infection (p value <0.05). (B) Quantitative RTPCR (q-RTPCR) validation of differentially expressed miRNAs. (C) Specific fold change (FC) values in differentially expressed miRNAs in HCV infected Huh7.5 cells ranked by p-value.

To assess the impact of acute HCV infection *in vitro* on changes in miRNA expression profiles, Huh7.5 cells were inoculated with 1.67×10^7^ copies of HCV genotype2a (JFH/J6) for 4 hours thereby establishing a dynamic and continuous HCV infection system. Total RNA isolated from four separate and consecutive cell passages was applied to the Human MicroRNA Panel (Applied Biosystems) containing 365 miRNA primers and probes (see Materials and Methods). After filtering non-specific signals below the threshold level, fold changes in miRNA expression were calculated using the comparative Ct method. Statistical analysis revealed a distinct miRNA signature between uninfected and HCV-infected Huh7.5 cells. Seven miRNAs were identified with a ≥1.3 fold decrease in expression levels at a p value ≤0.05 (Fig. 1A). Quantitative reverse transcription PCR (qRT-PCR) validated the miRNA expression results by confirming that expression patterns were consistent with the TaqMan® Array data (Fig. 1B). Of the 7 down-regulated miRNAs, miR-324-5p and miR-130a exhibited the greatest reduction in expression at a fold change (FC) of 3.50 (p = 0.0246) and 3.48 (p = 0.0110) respectively (Fig. 1C).

### Potential Effects of miRNA Down Regulation on HCV Entry and Replication *in vitro*

**Figure 2:**
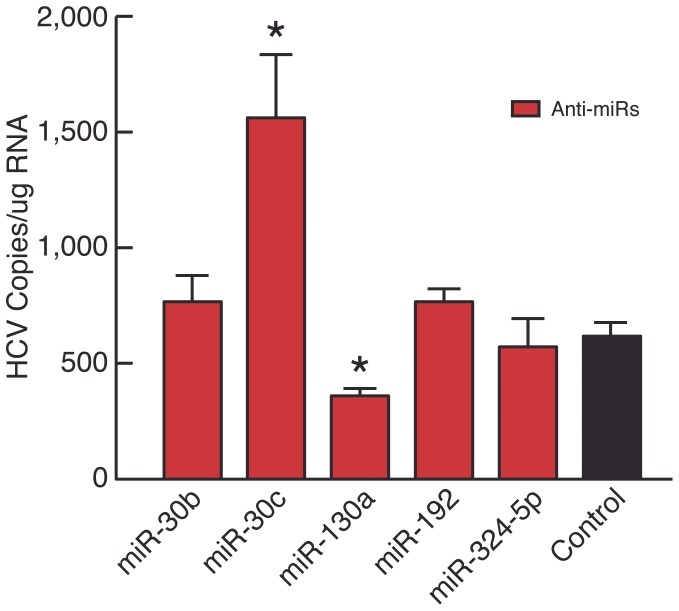
Impact of miRNA function on HCV replication in Huh7.5 cells. Five synthetic inhibitors (Ant-MiRs) for differentially regulated miRNAs and the miRNA inhibitor controls with no specific targets were transfected into naïve Huh7.5 cells then infected with HCV and analyzed for HCV replication. A Mann-Whitney test of multiple comparisons confirmed a significant increase in HCV RNA for Ant-miR-30c and significant decrease in HCV RNA for miR-130a. (*) P≤0.05.

Differential expression of host miRNAs may influence the complex pattern of interactions between viral replication and immunity-related mechanisms of anti-viral defense and viral immune evasion. To further investigate the potential effect of miRNA down regulation on HCV entry and replication *in vitro*, selected miRNA inhibitors (Anti-miRs) were synthesized, transfected into Huh7.5 cells, and infected with HCV. Seventy-two hours post-infection, HCV replication was quantitated by virus entry RT-PCR. Anti-miRs specific for 5 miRNAs down regulated upon HCV infection (miR-30b, miR-30c, miR-130a, miR-192, and miR-324-5p) were tested against a mock-transfected HCV^+^ Huh7.5 cell control (Fig. 2). Anti-miR controls with no specific target were used in each transfection (data not shown). Inhibition of miR-30c expression significantly enhanced HCV replication (951.5 HCV RNA copies, p value = 0.029) while miR-130a inhibition decreased HCV RNA compared to the HCV infected Huh7.5 control (352 mean HCV RNA copies, p value = 0.018). No effect on HCV replication was observed with miR-30b, miR-192 and miR-324-5p *in vitro*.

### Interferon-α Treatment Modulates Expression of HCV-specific miRNAs

**Figure 3:**
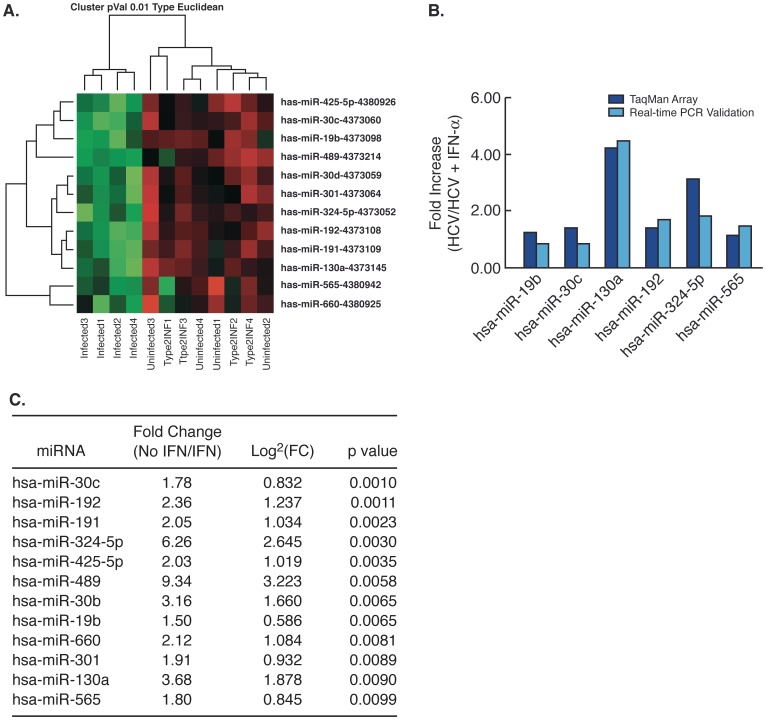
MiRNA expression and validation in HCV-infected Huh7.5 cells treated with IFN-α. (A) Heat map of differentially expressed in uninfected, HCV-infected, and HCV-infected with IFN-α, Huh7.5 cells. Twelve miRNAs were differentially expressed in response to IFN-α at a p value ≤0.01. (B) Q-RTPCR validation of 7 of the 12 differentially regulated miRNAs. (C) Specific fold changes in differentially expressed miRNAs ranked by p- value.

To determine the effect of IFN-α on acute HCV infection *in vitro*, RNA from uninfected and infected Huh7.5 cells with or without IFN-α treatment was applied to the Human TaqMan® Array. Statistical analysis revealed up-regulated levels of expression at a fold change greater than 1.5 for 12 miRNAs following IFN-α treatment (adjusted P value <0.01) (Fig. 3A). Quantitative RT-PCR validation of 7 out of 12 miRNAs confirmed that the expression patterns were consistent with the miRNA array profile (Fig. 3B). MiR-324-5p (FC = 6.26, p = 0.003) and miR-489 (FC = 9.34, p = 0.006) exhibited the greatest degree of up-regulation in the presence of IFN-α while miR-30c and miR-130a demonstrated the greatest difference in expression between HCV-infected Huh7.5 cells treated with or without IFN-α (Fig. 3C). Interestingly, 7 of the 12 miRNAs that were up-regulated in the presence of IFN-α (miR-30b,miR-30c, miR-130a, miR-192, miR-301, miR-324-5p and miR-565) were also down-regulated in HCV -infected Huh7.5 cells.

### Gene Ontology and Pathway Prediction of Differentially Expressed miRNAs

To determine if miRNAs might predict cellular or molecular changes in Huh7.5 cells upon HCV infection and IFN-α treatment, bioinformatic analysis using miRNA target prediction tools were applied to a subset of the 6 miRNAs that were differentially expressed during *in vitro* HCV infection with or without IFN-α. The GOMir tool JTarget, using five major miRNA-mRNA prediction databases, identified a list of mRNA targets for miR-30, miR-130a, miR-192, miR-301 and miR-324-5p [21]. Genes corresponding to mRNA targets were evaluated for biological relevance using the DAVID platform for predicting gene ontology, bio-pathways and gene function annotation. An EASE of 0.05 and Fisher exact p-value were used to organize each gene target list into functionally related groups. The most significantly enriched functional groups for the majority of miRNAs tested were found predominantly in the phosphoprotein, transcription, transcription regulation, nucleus, alternative splicing and cytoplasm functional categories (Fig. S1). The DAVID Pathway analysis tool applied to the same data sets revealed targeted bio-pathways for the miR-30 cluster (miR-30a, miR-30b, miR-30c and miR-30d) and miR-130a/301 cluster (Table 1). Two pathways, ubiquitin-mediated proteolysis and regulation of actin cytoskeleton, were predicted for MiR-30 at a p-value of 3.2×10^−3^ and 6.33×10^−3^ respectively. The fold enrichment for gene targets in the ubiquitin pathway was 2.12 compared to 1.78 fold for the regulation of actin cytoskeleton pathway. MiR-130a/301 exhibited significant gene enrichment within the TFG-β signaling pathway (p = 8.02×10^−6^), the endocytosis pathway (p = 2.94×10^−6^) and purine metabolism (7.24×10^−6^).

**Table 1:** Biological pathways targeted by miRNAs differentially expressed in HCV infection and following IFN-α treatment.

MiRNA	Biological Pathway	P value <0.05	Genes	Fold Enrichment	Benjamini	FDR
**30a/30b/30c/30d**	Ubiquitin mediated proteolysis	3.20E-03	SOCS3, UBE2G1,SOCS1, UBE2J1, BIRC6,HERC3, UBE21, UBE3C, MID1, UBE2R2, CUL2,UBE20, UBE2D3, CBLB, UBE2D2, NEDD4,UBE2K, WWP1, NEDD4L	2.12	1.14E-01	3.76
	Regulation of actin cytoskeleton	6.33E-03	GNA13, ENAH, SSH2, AB12, ITGB3, PXN,PFN2, KRAS, SOS1, PPP1R12A, PIK3R2,VAV3, ARHGEF6, PIK3CD, ACTN1,ITGA4, VAV2, CRKL,ITGA6, ITGA5,CFL2, PDGFRB, PIP4K2A, MYH10, PIP4K2B	1.78	1.48E-01	7.31
**130a/301**	Endocytosis	2.94E-06	RAB5B, LDLR, ERBB4, CHMP4B, ERBB3,VPS37A, VPS37B, KIT, CLTC, ZFYVE20,ACVR1C, GIT2, HSPA8, RAB4A, TGFBR1,TGFBR2, MET, PSD3, EPS15, PSD, RAB5A,VPS24, PDGFRA, RNF31, DNM2	2.93	4.00E-04	0.00
	TGF-β signaling pathway	8.02E-06	TNF, ROCK2, TGFBR1, SMAD5, TGFBR2,SMAD4, BMPR2, SMAD2, SKP1, ACVR1C,INHBB, MAPK1, SP1, ZFYVE9, BMPR1B,ACVR1	3.96	5.45E-04	0.01
	Purine metabolism	7.24E-06	ADCY3, ENPP1, PNPT1, PDE11A, POLR1C, PDE3A, PDE4D, GUCY2C, HPRT1, ADA, NME7, PFAS, PDE1B, PKM2, PKLR, PDE4B, RRM1, NT5C2, ADSL, PRPS1L1	3.29	1.09E-03	0.01

FDR = False Discovery Rate.

### Predicted mRNA Targets Correlate with miRNA-130a/301 Expression during HCV Infection and IFN Treatment *in vitro*

**Figure 4:**
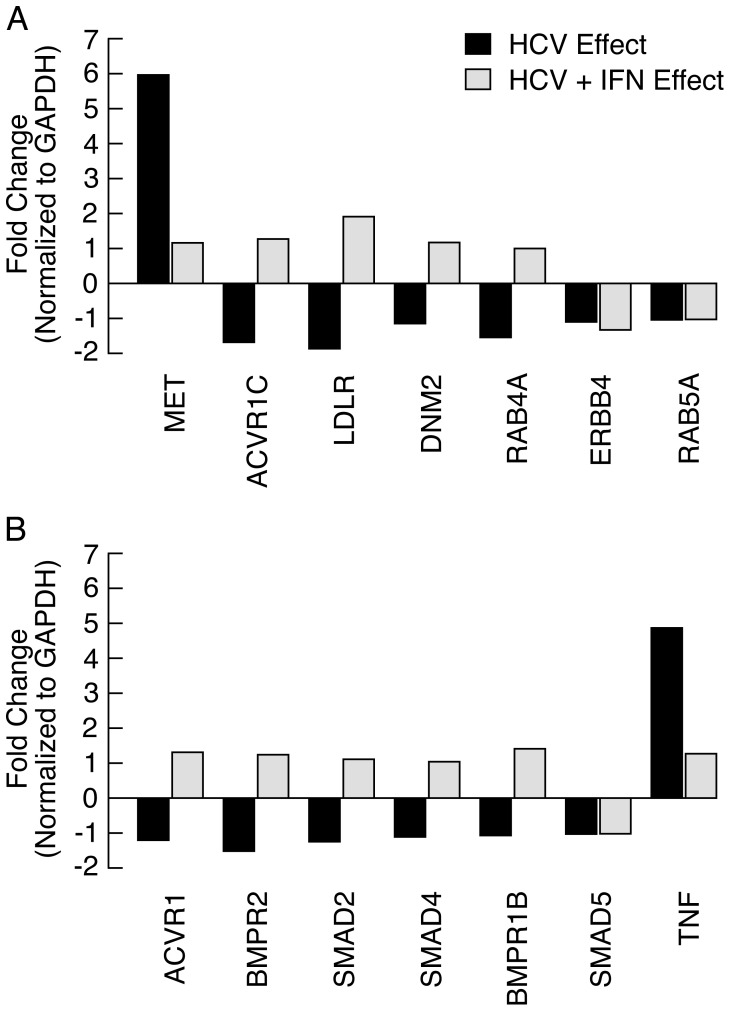
Expression of mRNA targets of miR-130a/301 in HCV-infected Huh7.5 treated with IFN-α. The top seven highly predicted mRNAs of gene targets for miR-130a/301 found in the endocytosis pathway (A) and TGF-β signaling pathways (B) were tested for expression using real-time quantitative RT-PCR and HCV-infected Huh7.5 cells without IFN-α (HCV effect) and with IFN-α (HCV+ IFN effect). The fold change in gene expression is normalized to GAPDH.

Although bioinformatic analyses of miRNA-mRNA correlated pairs provides a framework to predict miRNA interactions in a cellular environment following HCV infection, the functional importance of the predicted miRNA/mRNA interaction needs to be validated. To test the association between selected miRNA-130a/301-mRNAs found using DAVID, real-time quantitative RTPCR was performed on 7 highly predicted mRNAs of gene targets from the endocytosis pathway and 7 highly predicted gene targets from the TGF-β signaling pathway (Table 1) in HCV-infected Huh7.5 cells without IFN-α or following a 24-hour treatment with IFN-α. Interestingly, 4 gene targets (Activin A Receptor Type 1 (ACVR1C), Low density lipoprotein receptor (LDLR), Dynamin (DNM2), and Ras oncogene family member RAB4A) exhibited similar patterns of expression with a reduction in HCV-infected cells and induction in HCV-infected cells treated with IFN-α (Fig. 4A). The gene target, c-MET showed a 5.96-fold increase in HCV infection that was reduced to 1.16 in the presence of IFN-α. Similar levels of expression were observed in HCV infected cells with or without IFN for the v-erb viral oncogene homolog, ERBB4, and RAB5A. The majority of mRNA targets correlated with miR-130a/301 and involved in TGF-β signaling exhibited expression levels that were reduced in the presence of HCV infection and subsequently increased following IFN-α treatment with the exception of SMAD5 and Tumor Necrosis Factor (TNF). TNF expression increased 4.8-fold in the presence of HCV (HCV Effect) and decreased following IFN-α treatment (HCV+IFN Effect) (Fig. 4B).

## Discussion

Our results demonstrate that several miRNAs are differently regulated by HCV infection and IFN-α treatment in human hepatoma cells *in vitro* suggesting a different host response to both HCV and IFN-α. In this regard, several miRNAs that were down regulated by in HCV infection of Huh7.5 cells were up-regulated following treatment of infected cells with interferon. These differentially expressed miRNAs are reciprocally correlated with mRNAs of key genes associated with cellular pathways that may make hepatocytes more permissive to HCV entry and replication. Moreover, expression of these miRNAs is altered upon treatment with interferon. MiRNAs control multiple biological and metabolic processes, ranging from developmental timing, organ and tissue development and signal transduction to diseases including cancers and infectious diseases. The liver-specific miR-122 is the first host miRNA that has been shown to exert a positive effect on HCV replication in cell culture by binding in the viral 5′ UTR [25]. Recent reports further demonstrated that the *in vivo* steady level of miR-122 correlates with the HCV responsiveness to interferon therapy [26]. Moreover, several interferon-modulated cellular miRNAs were characterized to target the HCV genome and show significant antiviral effects in a sequence-specific manner [10] [27]. Other miRNAs such as miR-196 and miR-199a have been shown to down regulate HCV in Con1 and JFH1 cell culture systems or directly modulate the interferon response to HCV infection in mammalian organisms [9] [28]. Collectively, these findings highlight the important role of host miRNAs in regulating liver-specific HCV replication and pathogenesis and suggest that miRNAs might be potential biomarkers for predicting interferon response to HCV infection.

Our data suggest that the miR-30(a–d) cluster and miR130a/301are significantly associated with gene targets found in pathways that involve HCV entry and replication, and thus, may play a role in the pathogenesis of chronic liver disease. Loss of function experiments using anti-miRs revealed that inhibition of miR-30c increased HCV RNA levels *in vitro*. Bioinformatic analysis predicted that mRNAs of gene targets associated with the miR-30 cluster were concentrated in 2 major pathways – ubiquitin mediated proteolysis and regulation of actin cytoskeleton (Fig. S2, Fig. S3). Several miR-30 targets including the Suppressor of cytokine signaling 1 and 3 (SOCS1, SOCS3) genes contained conserved 8 mer sites that matched the seed region of miR-30c (Table 2). SOCS proteins comprise a family of intracellular proteins that act as negative regulators of cytokine signaling. Specifically, SOCS1 and SOCS3 inhibit JAK tyrosine kinase activity and STATs in the JAK-STAT signaling pathway play a role in ubiquitination through interactions with elongin B and elongin C, Cullins, and the RING-finger domain-only protein RBX2 (reviewed by [29].

**Table 2:** Location of putative miR-30c binding sites in the 3′ UTR of target mRNAs.

Ubiquitin-Mediated Proteolysis				
mRNA-miRNA Pair	Predicted pairing of target and miRNA	Position in 3′ UTR	Seed Match	P_CT_
**SOCS3**	5′ CUUGUUUUUUAAUAA**UGUUUAC**A	1425–1432	8 mer	0.93
*miR-30c*	3′ CGACUCUCACAUCCU**ACAAAUG**U			
**CUL2**	5′ GCCCCUGUUUUCUGC**UGUUUAC**A	121–128	8 mer	0.93
*miR-30c*	3′ CGACUCUCACAUCCU**ACAAAUG**U			
**NEDD4**	5′ GUAGUAAAUGUA–**UGUUUAC**A	942–949	8 mer	0.93
*miR-30c*	3′ CGACUCUCACAUCCU**ACAAAUG**U			
**SOCS1**	5′ CCUCCUACCUCUUCA**UGUUUAC**A	285–292	8 mer	0.91
*miR-30c*	3′ CGACUCUCACAUCCU**ACAAAUG**U			
**Regulation of Actin Cytoskeleton**				
**mRNA-miRNA Pair**	**Predicted pairing of target and miRNA**	**Position in 3′ UTR**	**Seed Match**	**P_CT_**
**ITGB3**	5′ GAGUCCCUGCCAUCA**UGUUUAC**A	65–72	8 mer	0.92
*miR-30c*	3′ CGACUCUCACAUCCU**ACAAAUG**U			
**ARHGEF6**	5′ GCCAUAGU**UGU**GU–**UGUUUAC**A	2292–2299	8 mer	0.91
*miR-30c*	3′ CGACUCUC**ACA**UCCU**ACAAAUG**U			
**ITGA4**	5′ AAAUUUAAAAGACAC**UGUUUAC**A	67–74	8 mer	0.84
*miR- miR-30c*	3′ CGACUCUCACAUCCU**ACAAAUG**U			
**PIK3R2**	5′ UGGCUGCACCUGCCA**UGUUUAC**A	371–378	8 mer	0.83
*miR-30c*	3′ CGACUCUCACAUCCU**ACAAAUG**U			

P_CT_ – Probability of conserved targeting (Friedman, 2009).

mRNAs of gene targets associated with regulation of actin cytoskeleton include the Integrins beta 3 (ITGB3) and alpha 4 (ITGA4). Integrins are a large family of heterodimeric cell surface receptors, which act as mechanoreceptors by relaying the information from cell to cell and from the extracellular matrix (ECM) to the cell interior and vice versa [30]. Although integrins play an important role in the development of liver fibrosis, cholestatic liver diseases and inflammation, their involvement in viral hepatitis remains poorly understood. An interesting link between integrins and HCV was found by sequence analysis of the HCV non-structural protein 3 (NS3) identifying an RGD (Arginine-glycine-aspartic acid) motif thought to promote integrin-mediated cell adhesion within a region of high hydrophobicity and cell surface probability. Data showed that NS3 may disturb the migration of immune cells to infected cells by blocking the RGD-binding site, thus protecting infected cells from the cellular immune response [31]. We hypothesize that the down regulation of miR-30 in HCV-infected Huh7.5 cells may alter the expression of genes involved in the ubiquitin and actin cytoskeleton pathways that create a permissive environment for viral replication and this “proviral effect” is diminished with the addition of IFN-α.

We found the reverse HCV effect following the inhibition of miR-130a/301 expression. HCV copy number was significantly reduced compared to the control. The mRNAs of targeted genes for miR-130a/301 were found in the endocytosis and TGF-β signaling pathways (Fig. S4, Fig. S5). We confirmed the expression of 7 gene targets from each pathway and found differential expression patterns in HCV-infected cells with and without IFN-α. Of note was the high expression of the proto-oncogene, c-Met. C-Met, also known as the hepatocyte growth factor (HGF) receptor, has been implicated in the development of hepatocellular carcinoma [32]–[33]. IFN-α suppresses c-Met promoter activity through the binding of Sp1 which may contribute to the antitumor activity of IFN-α in the liver [34]. Our data suggests that, in addition to possible antitumor effects, treatment of HCV-infected Huh7.5 cells with IFN-α upregulates miR-130a/301 thereby reducing c-Met expression and HCV pathogenesis. There is a significant probability that the predicted seed match of miR130a/301 binds to the 3′ UTR of MET (Table 2), but there is no direct evidence that the differential expression of miR-130a/301 results in an anti-viral effect. Further characterization of mRNAs of gene targets in the endocytosis pathway may reveal the mechanism(s) associated with reduced HCV replication in infected Huh7.5 cells in the absence of miR-130a/301. Similarly, within the TGF-β pathway, several genes were expressed that potentially affect HCV replication and IFN-α response. In the liver, during HCV infection, TGF-β is responsible for hepatocyte regeneration and fibrosis, and for epithelial cell proliferation and differentiation. TGF-β concentrations are higher the more severe the liver failure is and subsequently decreases in patients with chronic hepatitis C following successful antiviral therapy. An important component of TGF-β signaling in hepatitis infection consists of the SMAD family of proteins. SMADs modulate the activity of TGF-β ligands through a complex array of protein-protein interaction in the cytoplasm and the nucleus that result in increased transcription and apoptosis. Whether there is a direct association between SMADs and miRNAs remains to be clarified. Predicted binding of miR-130a/301 seed sequences to the 3′UTR of SMAD4 and SMAD5 was highly probable (Table 3) but not at the level of other gene targets identified in the TGF-β signaling pathway.

**Table 3:** Location of putative miR-130a binding sites in the 3′ UTR of target mRNAs.

Endocytosis Pathway				
mRNA-miRNA Pair	Predicted pairing of target and miRNA	Position in 3′ UTR	Seed Match	P_CT_
**MET**	5′ UUGCUCUUGCCAAAA**UUGCACU**A	100–107	8 mer	0.90
*miR-130a*	3′ UACGGGAAAAUUGU**AACGUGA**C			
**RAB5A**	5′ GAUCAGUUGAGUAUA**UUGCACU**A	1162–1169	8-mer	0.74
*miR-130a*	3′ UACGGGAAAAUUGU**AACGUGA**C			
**DNM2**	5′ GGGGGCGCUGGGGUG**UUGCACU**U	224–230	7 mer-m8	0.76
*miR-130a*	3′ UACGGGAAAAUUGU**AACGUGA**C			
**LDLR**	5′ AUUCCCGUGGUCUCC**UUGCACU**U	871–877	7 mer-m8	0.59
*miR-130a*	3′ UACGGGAAAAUUGU**AACGUGA**C			
**TGF-β Signaling Pathway**				
**mRNA-miRNA Pair**	**Predicted pairing of target and miRNA**	**Position in 3′ UTR**	**Seed Match**	**P_CT_**
**ACVR1**	5′ UCUCUUC**UUUA**–**UUGCACU**A	419–426	8 mer	0.84
*miR-130a*	3′ UACGGGA**AAAU**UGU**AACGUGA**C			
**BMPR2**	5′ UUUGUUUUUUAAGUU**UUGCACU**U	204–210	7 mer-m8	0.89
*miR-130a*	3′ UACGGGAAAAUUGU-**AACGUGA**C			
**SMAD5**	5′ CAUUAAUCUUUUAUU-**UUGCACU**U	846–852	7 mer-m8	0.88
*miR-130a*	3′ UACGGGAAAAUUGU**AACGUGA**C			
**SMAD4**	5′ GAUUUUU**UUUUU**CUU**UUGCACU**U	1162–1169	7 mer-m8	0.78
*miR-130a*	3′ UACGGG**AAAAU**UGU**AACGUGA**C			

P_CT_ – Probability of conserved targeting [23].

The exact mechanism by which HCV down regulates miRNA or how miRNA suppresses HCV replication is presently not well known. In this regard, a recent study suggested that miR-130 mediates suppression of HCV replication by inducing the expression of Interferon stimulating gene (ISG), IFITM3 [35]. Although we did not observe an overexpression of this gene in our system, similar candidate genes may mediate this effect. Ongoing studies in the laboratory are aimed at looking at various stages of HCV replication in Huh7.5 cells with or without high levels of individual miRNA. Overall, this study suggests that differential regulatory miRNA expression pathways induced *in vitro* by HCV and interferon-α offer a novel mechanism for investigating disease pathogenesis and biologic response to HCV therapy.

## Supporting Information

Figure S1 **Gene function prediction of differentially expressed miRNAs in HCV infection and response to IFN-α.** Bioinformatic tools JTarget and DAVID predicted miRNA gene targets and gene function respectively. MiRNA gene targets from at least 6 prediction programs (Tarbase, TargetScan, miRanda, RNAhybrid and PicTar-4way and PicTar-5way) created one gene list per miRNA. Each miRNA target list was entered into DAVID for gene functional analysis using an EASE of 0.05. The Fisher exact test was applied to all miRNA –associated gene functions and ranked by significance. P values ranged from 1.0E-48 (dark blue) to 5.0E-3 (light gray).
(TIF)

Figure S2 **MiR-30(a–d)-associated gene targets in the Ubiquitin-Mediated Proteolysis pathway.**
(TIF)

Figure S3 **MiR-30(a–d)-associated gene targets in the Regulation of Actin Cytoskeleton pathway.**
(TIF)

Figure S4 **MiR-130a-associated gene targets in the TGF-β signaling pathway.**
(TIF)

Figure S5 **MiR-130a-associated gene targets in the Endocytosis pathway.**
(TIF)
