# Supplementary figures and images for: MicroRNA Expression Profiling in HCV-Infected Human Hepatoma Cells Identifies Potential Anti-Viral Targets Induced by Interferon-α
Source: PLoS One. 2013 Feb 13;8(2):e55733. doi: 10.1371/journal.pone.0055733 (PMC3572124; doi:10.1371/journal.pone.0055733)

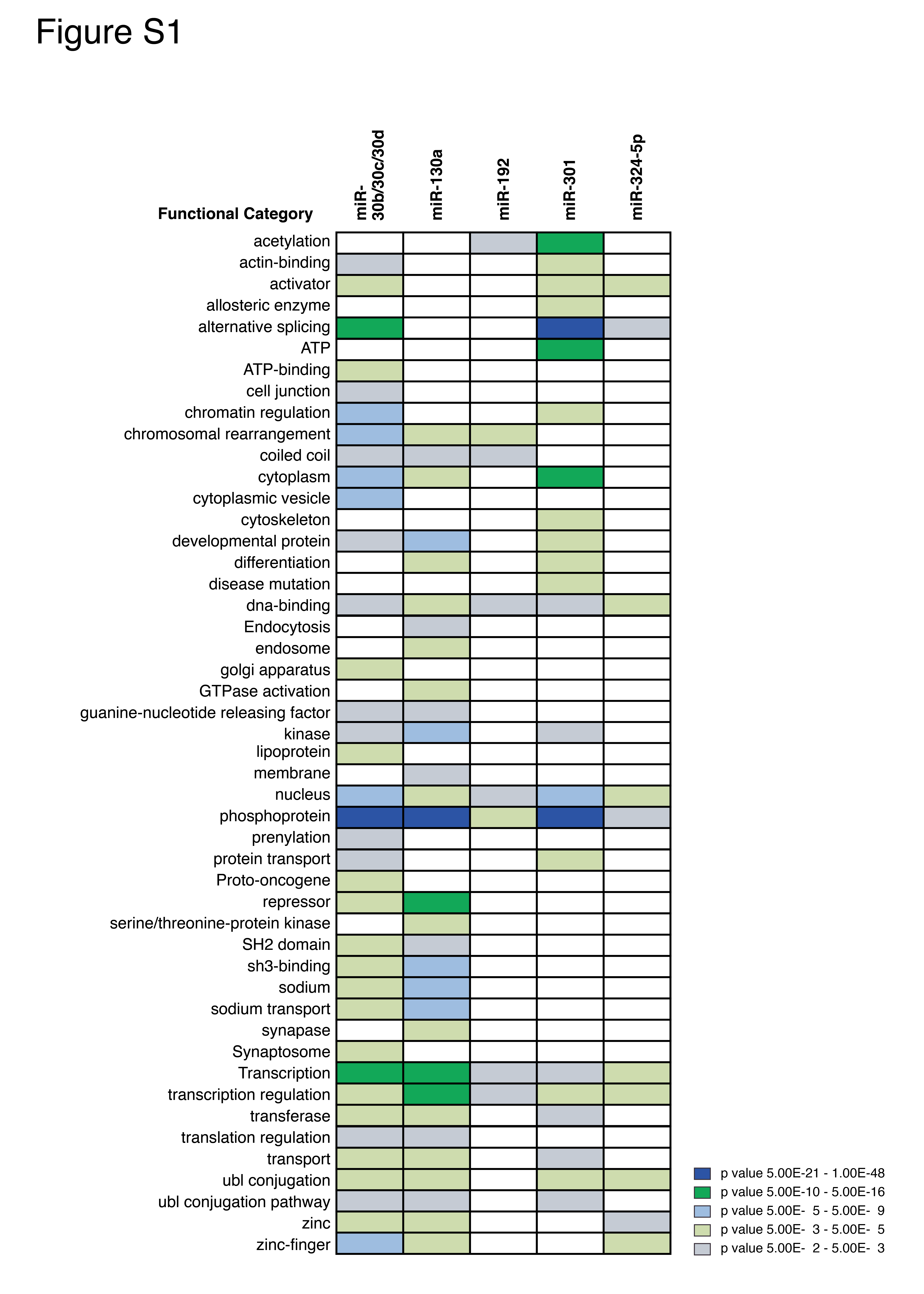

Supplement: Figure S1 — Gene function prediction of differentially expressed miRNAs in HCV infection and response to IFN-α. Bioinformatic tools JTarget and DAVID predicted miRNA gene targets and gene function respectively. MiRNA gene targets from at least 6 prediction programs (Tarbase, TargetScan, miRanda, RNAhybrid and PicTar-4way and PicTar-5way) created one gene list per miRNA. Each miRNA target list was entered into DAVID for gene functional analysis using an EASE of 0.05. The Fisher exact test was applied to all miRNA –associated gene functions and ranked by significance. P values ranged from 1.0E-48 (dark blue) to 5.0E-3 (light gray). (TIF) [file pone.0055733.s001.tif]

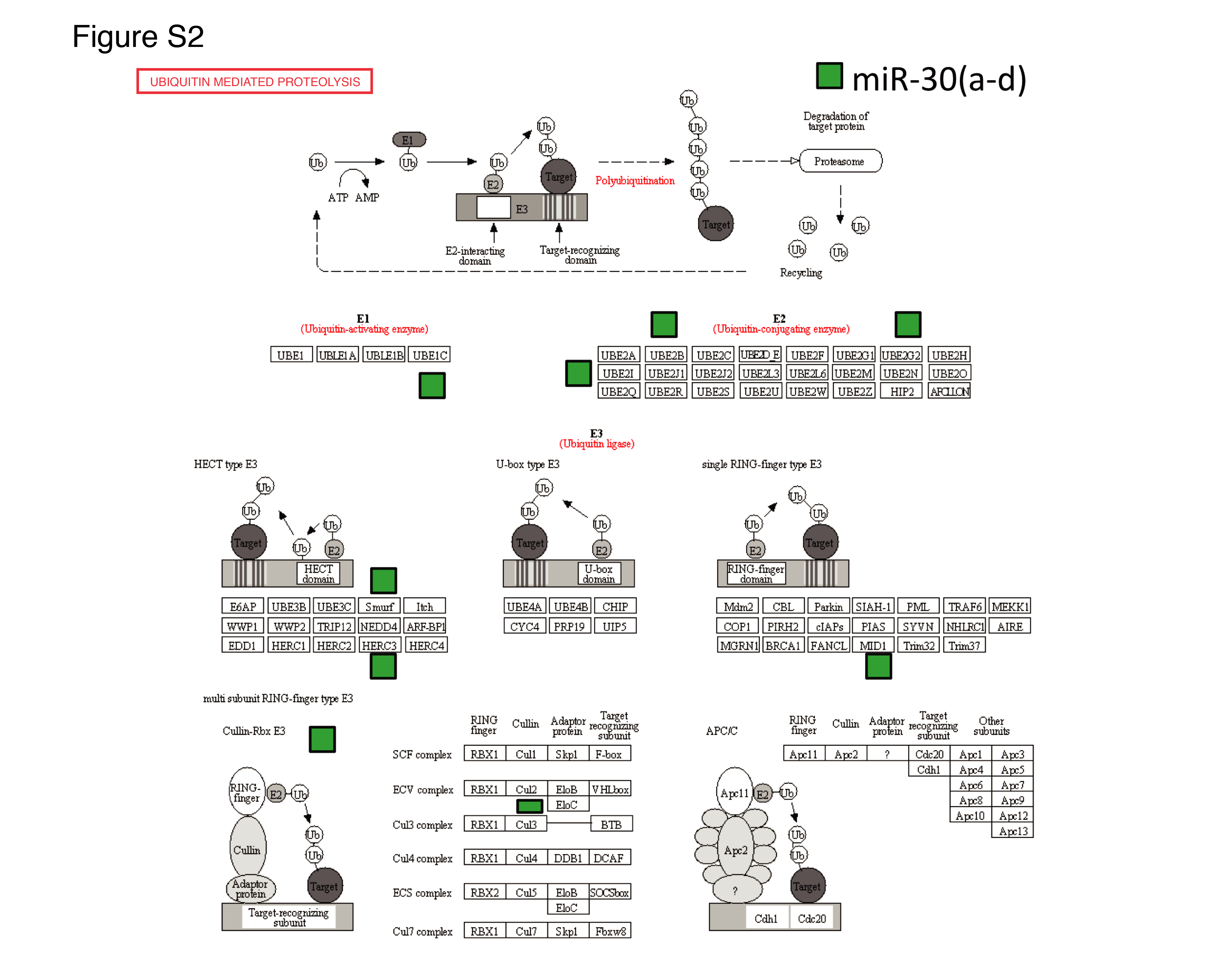

Supplement: Figure S2 — MiR-30(a–d)-associated gene targets in the Ubiquitin-Mediated Proteolysis pathway. (TIF) [file pone.0055733.s002.tif]

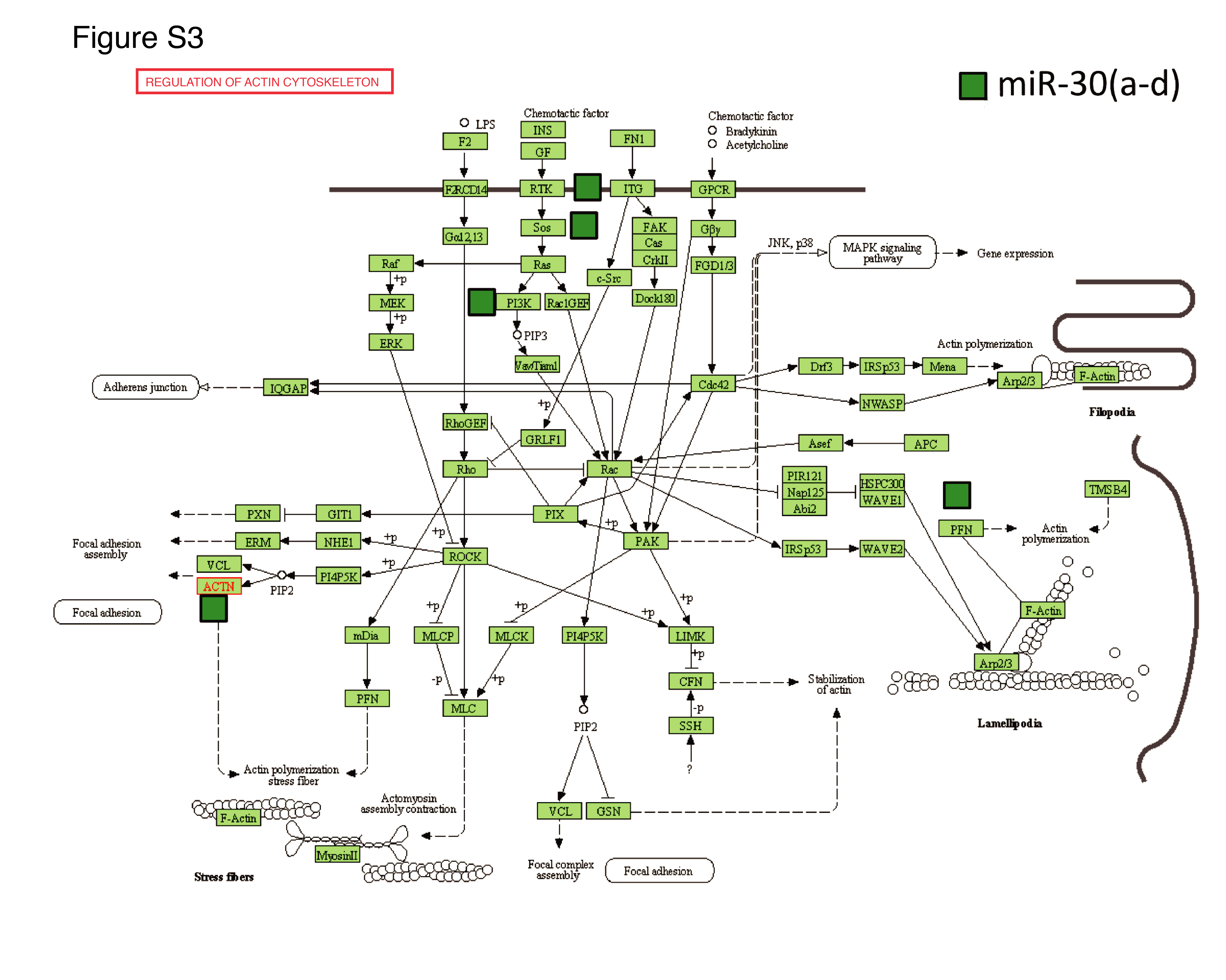

Supplement: Figure S3 — MiR-30(a–d)-associated gene targets in the Regulation of Actin Cytoskeleton pathway. (TIF) [file pone.0055733.s003.tif]

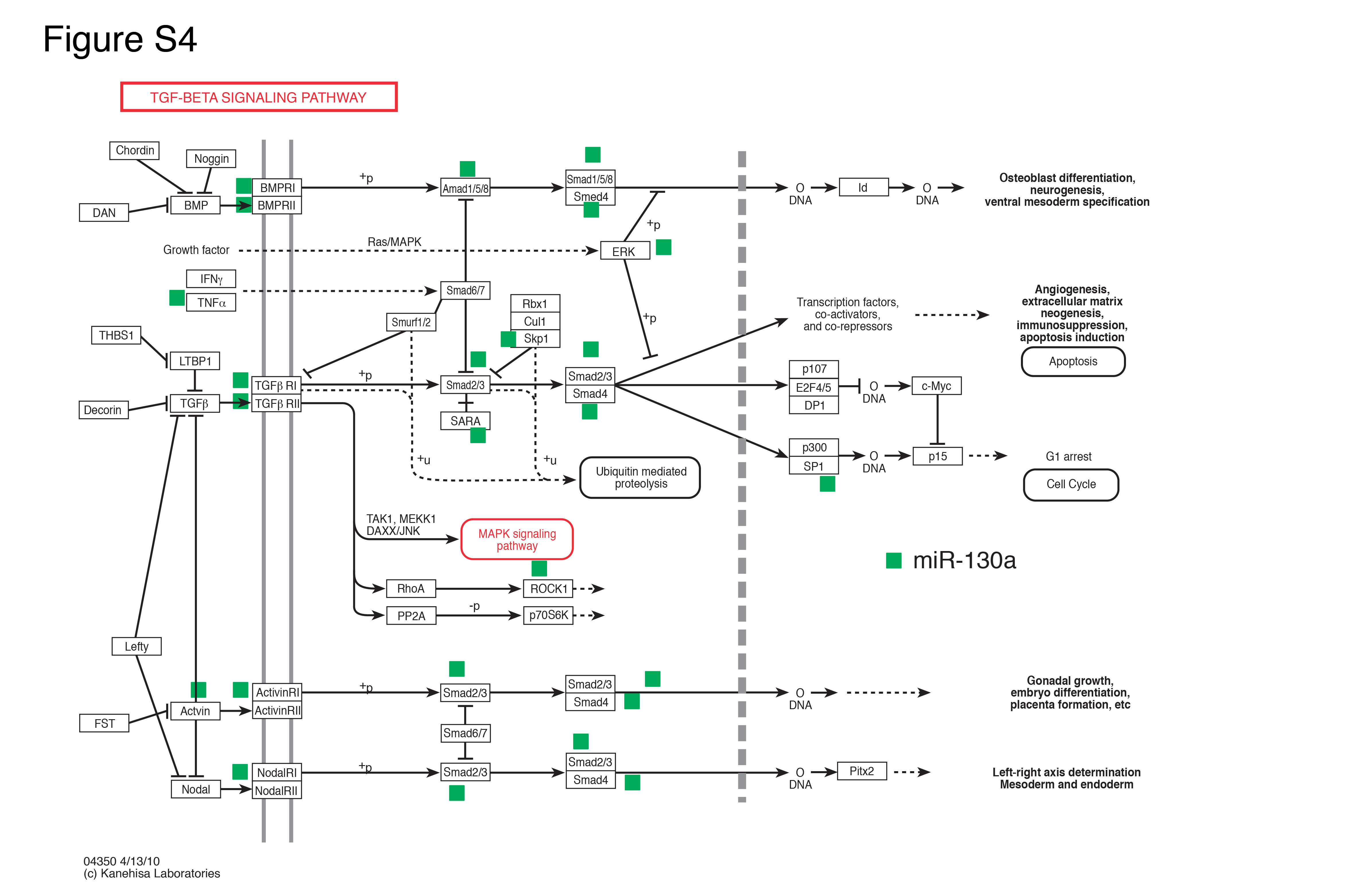

Supplement: Figure S4 — MiR-130a-associated gene targets in the TGF-β signaling pathway. (TIF) [file pone.0055733.s004.tif]

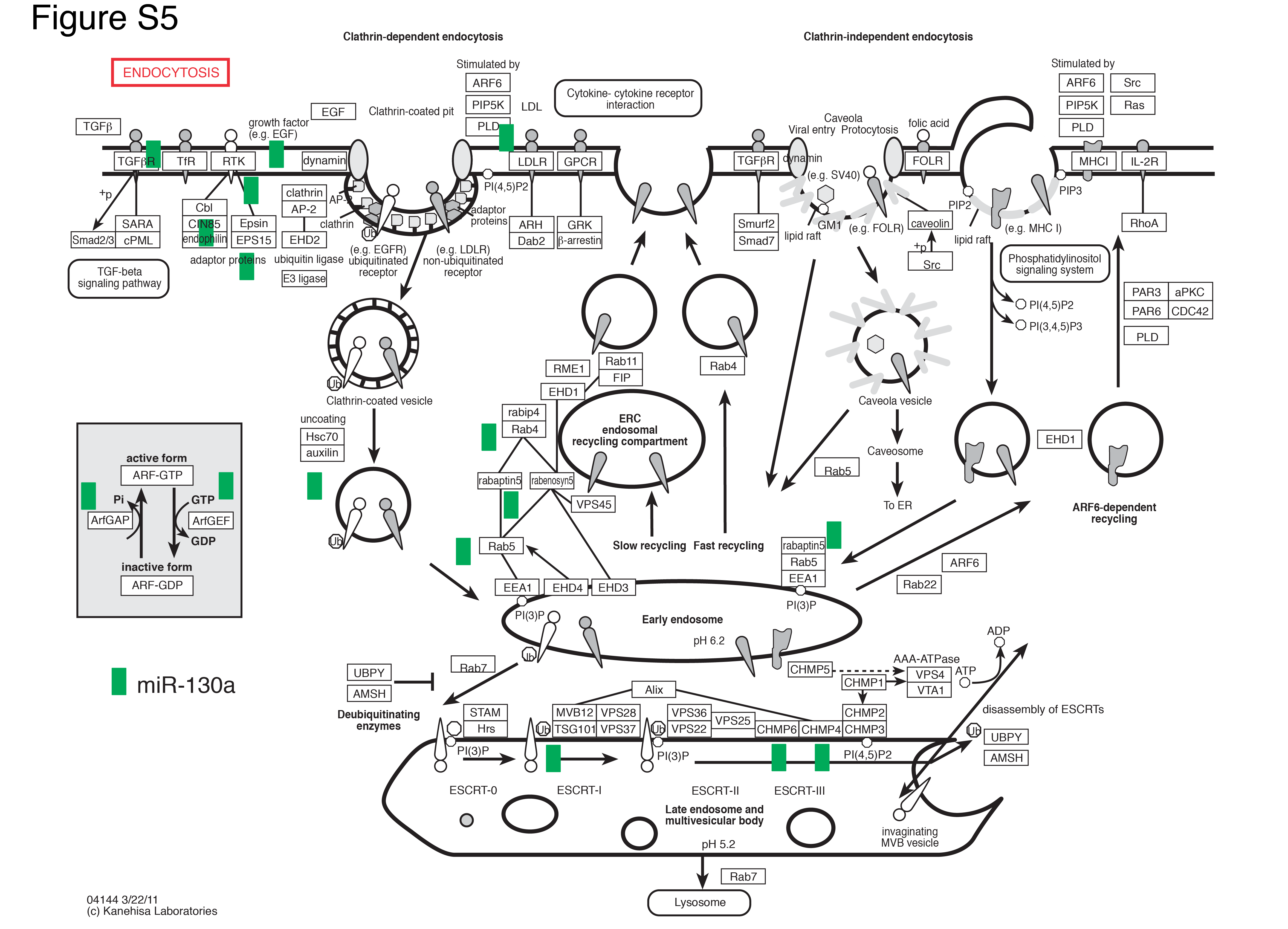

Supplement: Figure S5 — MiR-130a-associated gene targets in the Endocytosis pathway. (TIF) [file pone.0055733.s005.tif]
